# Hydatid cyst involving the mandible ramus

**DOI:** 10.4322/acr.2023.437

**Published:** 2023-06-22

**Authors:** Nivedhitha Maraimalai, Manisha Ahire Sardar, Kavita Wadde, Om Kharat, Shaheen Kanpurwala, Asha Chowdhar

**Affiliations:** 1 Government Dental College and Hospital, Department of Oral Pathology and Microbiology, Mumbai, India; 2 Government Dental College and Hospital, Department of Oral and Maxillofacial Surgery, Mumbai, India; 3 Government Dental College and Hospital, Department of Oral Medicine and Radiology, Mumbai, India; 4 Government Dental College and Hospital, Department of General Pathology, Mumbai, India

**Keywords:** Echinococcosis, Hydatidosis, Hydatid Cysts, Jaw Cysts

## Abstract

Parasitic infections rarely involve the oral and maxillofacial regions and pose a diagnostic challenge when they do. Hydatid cysts are parasitic cysts caused by *Echinococcus granulosus*. Intraosseous involvement is observed in 3% of cases, of which only 2-6% are in the maxillofacial region. A scientific literature search revealed only seven cases involving the mandible. We report a rare case in a 16-year-old female patient who presented with facial asymmetry and well-defined radiolucency of the ramus. Our findings will help in understanding the diagnostic issues caused by non-specific presentation and difficulties in suspecting such a rare diagnosis as echinococcosis of the oral or maxillofacial region. A thorough systemic investigation is essential as 20-30% of these cases show multiorgan involvement.

## INTRODUCTION

Hydatid cysts, also known as human cystic echinococcosis, have been reported since time immemorial. The word “Hydatid” in Greek translates to “a drop of water”.^1^ Hydatid cysts most commonly affect the liver and lungs. Fortunately, intraosseous involvement is sporadic, accounting for only 3% of all cases. Mandibular involvement is infrequent, with seven cases reported in the literature to date.^2^

## CASE REPORT

A 16-year-old female patient visited our institute with a chief complaint of swelling on the left side of her face for 1 month. The swelling presented gradual and slow growth and became more evident over the last month. Medications prescribed by a local facility did not resolve the swelling. The patient denied any history of trauma.

**Figure 1:**
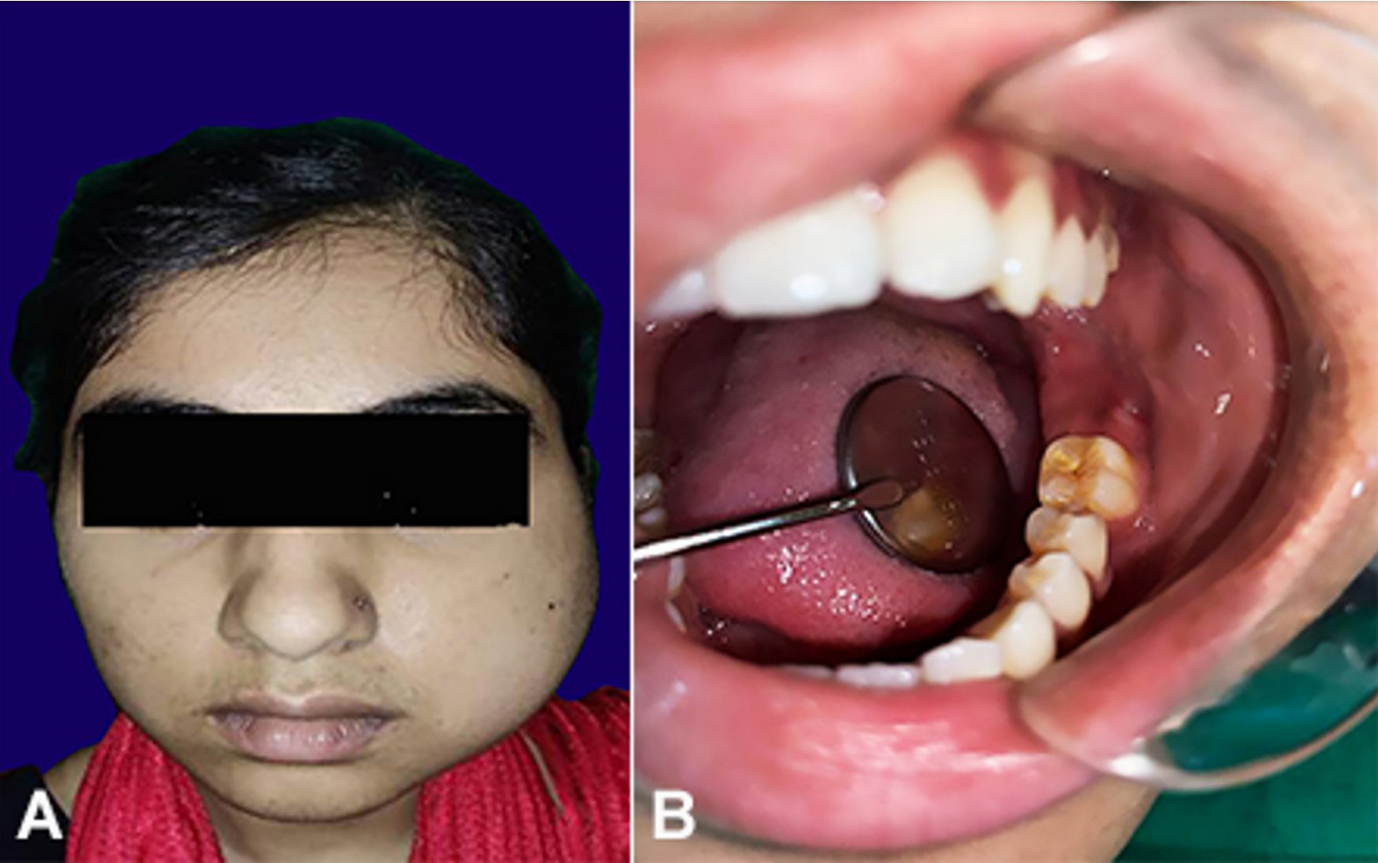
Gross view of: **A -** facial asymmetry and extraoral swelling evident along the left side of the face; **B -** draining sinus with pus discharge evident at the left mandibular third molar region.

Extraoral examination revealed non-tender and diffuse expansile swelling on the left side of the face, extending from the outer canthus of the eye to the lower border of the mandible and from the malar region to the posterior border of the mandible on the left side. The patient had a mouth opening of 5 mm. Intraorally, obliteration of the buccal vestibule was noted in relation to left mandibular molar region. A draining sinus with pus discharge was evident at the left mandibular third molar region (Figure 1A, and 1B).

**Figure 2:**
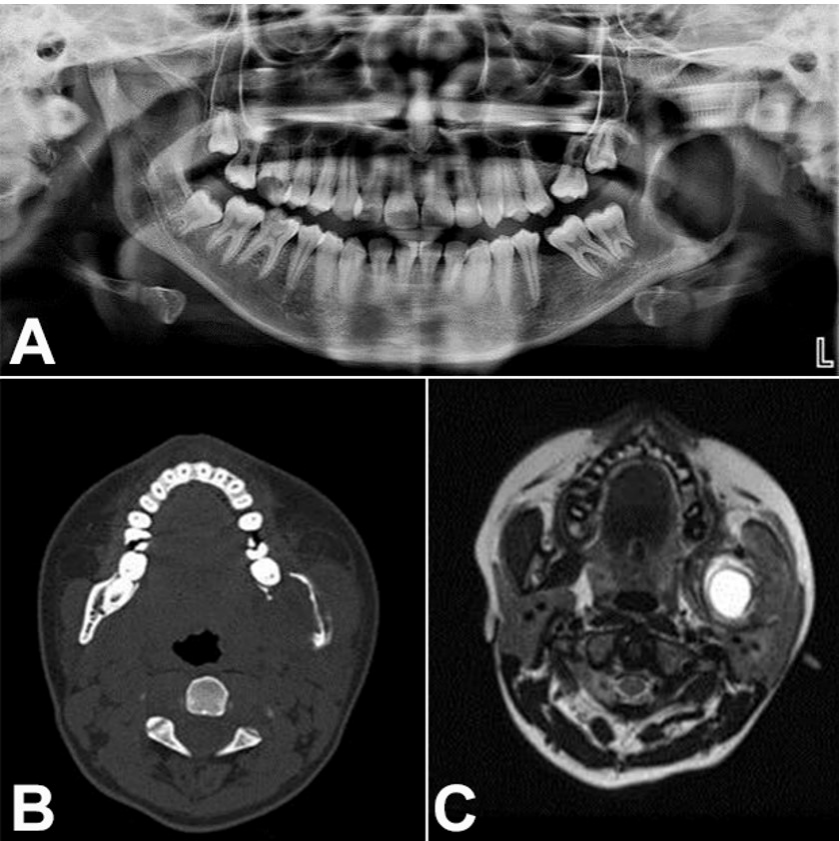
**A -** Orthopantamogram shows well-defined radiolucency along the left ramus region; **B –** Computed tomography axial view shows a well-defined, expansile, destructive lesion with perforation of the buccal and lingual cortical plate; **C –** Magnetic Resonance Imaging T2-weighted axial images show hyperintense signal with well-defined capsule surrounding the lesion in the posterior ramus of the left mandible.

OPG revealed well-defined radiolucency along the left mandibular ramus (Figure 2A). CT axial view showed a well-defined, expansile, destructive lesion with perforation of the buccal and lingual cortical plate. MRI T2-weighted axial images revealed a hyperintense signal with a well-defined capsule surrounding the lesion in the posterior ramus of the left mandible (Figure 2B and 2C).

Based on the clinical and radiological features, a provisional diagnosis of an infected odontogenic keratocyst, orthokeratinized odontogenic cyst, and unilocular ameloblastoma was made.

An incisional biopsy, along with the extraction of the left mandibular third molar, was performed under local anesthesia.

**Figure 3:**
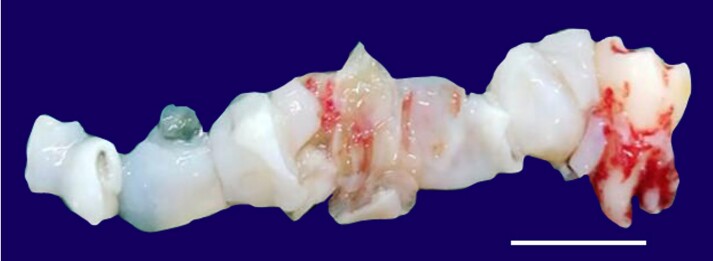
Gross view of the biopsy. Scale bar = 2 cm.

Gross examination of the incised specimen revealed multiple bits of whitish to yellowish-white, soft, membranous gelatinous tissue resembling tender coconut meat of varying sizes (Figure 3).

**Figure 4:**
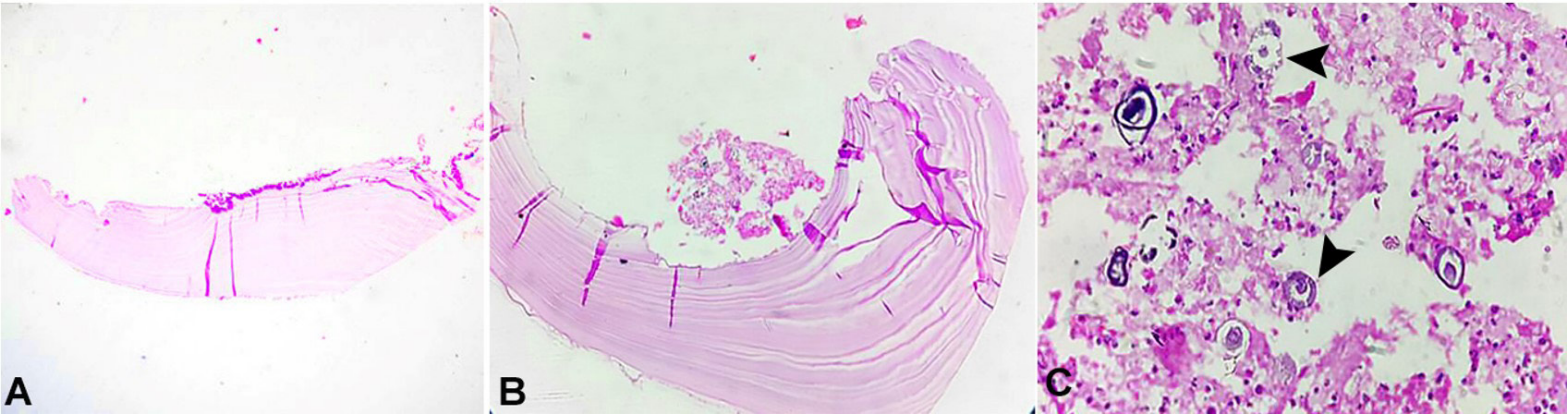
Photomicrograph of the biopsy. **A** and **B -** acellular, avascular, eosinophilic lamellated tissue suggestive of chitin (H&E, 4× and 10×, respectively); **C -** protoscolices (arrowheads) in the germinal membrane (H&E, 40×).

On histopathological examination, the H&E-stained sections showed multiple acellular, avascular, and eosinophilic lamellated structures consistent with chitin. These structures were lined with a germinal membrane from which the brood capsules developed. Detached germinal membrane with protoscolices of parasite form the “hydatid sand” was evident, which is pathognomonic of hydatid cyst (Figure 4A, 4B, and 4C).

The patient underwent further investigation to rule out multiorgan involvement. The abdominal and pelvic USG revealed a well-defined hyperechoic lesion measuring 8 × 9 mm in segment VIII of the right hepatic lobe, consistent with “calcified granuloma.” Other abdominal organs appeared normal. Echinococcus IgG ELISA was performed, which was reported to be equivocal. Casoni's intradermal test revealed positive findings. The patient was referred to a general surgeon for the management of liver hydatidosis.

**Figure 5:**
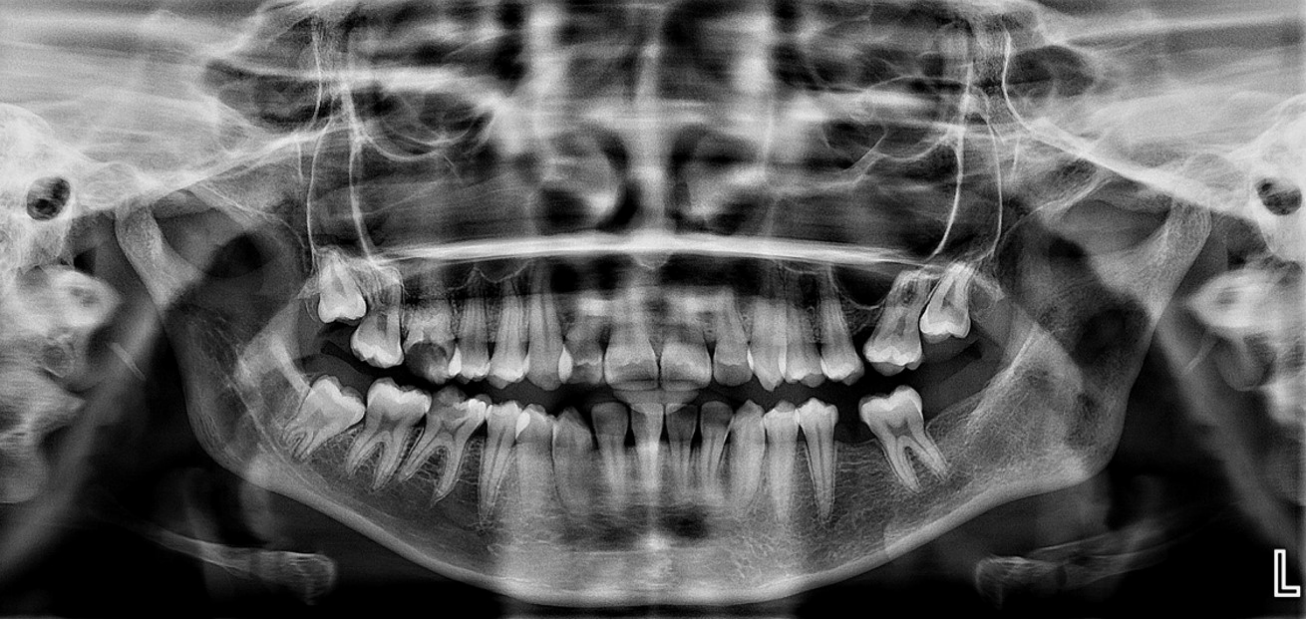
1-year follow-up with postoperative Orthopantamogram reveals a healing lesion with well-defined bony trabeculae formation.

Once the diagnosis was established, the patient was started on scolicidal therapy with albendazole 400 mg twice daily, with a scheduled treatment course for 2 years. The definitive surgical treatment comprised cystectomy through an intraoral approach under local anesthesia, and the remaining cyst tissue was removed. The incision healed without any dehiscence. The patient was followed up at regular intervals of 1 month, 3 months, 6 months, and 1 year, with no clinical symptoms. After a 1-year follow-up, OPG revealed a healing lesion with well-defined bony trabeculae formation and no evidence of recurrence (Figure 5).

## DISCUSSION

Hydatid cysts are benign, asymptomatic cystic lesions caused by the parasite *Echinococcus granulosus*. The incubation period is highly variable. Infection is most likely to occur during childhood and manifest clinically at later stages of life.^3^ The clinical and radiographic presentation is non-specific and depends upon the involved organ, size, and its effect on the surrounding tissues.^4^ Men and women are equally affected.^2,3^

**Figure 6:**
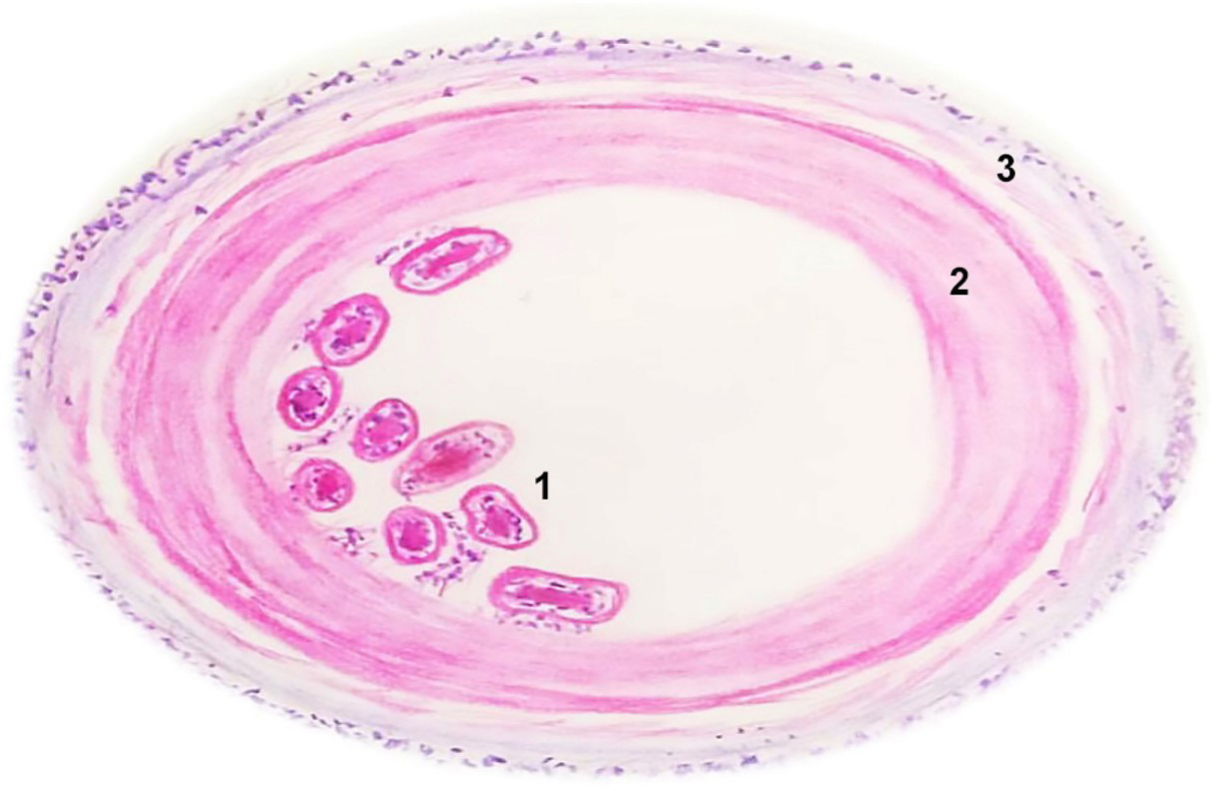
Diagrammatic representation of the three layers of hydatid cyst. **1 -** endocyst: comprising the germinal membrane along with protoscolices; **2 -** exocyst: comprising lamellated eosinophilic acellular avascular structures (chitin); **3 -** pericyst: fibrocellular tissue of the host comprising inflammatory cells and collagen fibers (diagram drawn by Nivedhitha M).

Microscopic examination revealed three layers. The innermost layer (endocyst) and intermediate layer (exocyst) are parasitic layers composed of protoscolices and hooklets in the hydatid sand and avascular eosinophilic lamellated structures, respectively. The outermost layer (pericyst) is the host layer, consisting of fibrous tissue and chronic inflammatory cell infiltrates. Figure 6 shows a schematic representation of a microscopic image of the hydatid cyst. The thickness of the layer depends on the location of the lesion. The thickest layers are found in the liver, whereas the thinner layers are noted in the muscles. No pericysts are noted in the bone.^5-7^ Similarly, the present case did not show pericysts because of intraosseous involvement.

Dogs and cattle are the primary and intermediate hosts, respectively. Humans are accidental hosts of this parasite. It is known to commonly affect the cattle-raising population.^8^ On inquiry, the patient reported a history of raising goats at home. The larval form of the parasite is mostly transmitted through ingesting contaminated food. Inside the body, the larva breaks down and releases an embryo that settles in the organs. Six to ten days after settling, the embryo becomes vacuolated. Intraosseous involvement is predominantly secondary to primary infection due to the seeding of the embryo on the bone. Subsequently, they produce microvesicles by exogenous budding and spread along the path of least resistance, where the daughter vesicles invade the bone and replace the medullary tissue. Ischemia and direct local pressure play an important role in bone destruction. Intraosseous cysts generally do not calcify. Over time, subsequent destruction of the bone cortex occurs with the dissemination of the disease to the surrounding tissues.^2,4^

Overall, 20-30% of patients with hydatid cysts show multiorgan involvement. The liver (50-70%) is the most commonly affected organ, followed by the lungs (20-30%), spleen (0.7- 8%), kidney (7%), muscles (4%), central nervous system (0.2-3%), and heart (0.2-2%).^8^ Intraosseous involvement is seen in <3% of cases. Furthermore, 2-6% of the total intraosseous cases affect the maxillofacial region.^2^ Hence, along with an ultrasound examination of the liver, chest radiography and MRI of the abdomen and brain are necessary to exclude the involvement of other organs. Serological investigations include IgG ELISA, indirect hemagglutination, latex agglutination, and immunoelectrophoresis.^3^ However, these tests have a high chance of false-negative results in intraosseous involvement and calcified cysts.^2^ The present case showed indefinite results in IgG ELISA, likely due to intraosseous involvement in the mandible and calcified granuloma in the liver.

Intraosseous hydatid cysts are confirmed only by biopsy, particularly if there is no evidence of the involvement of other organs. Once the histopathological diagnosis is confirmed, the treatment of choice for hydatid cysts of the maxillofacial region is surgical excision of the entire cyst in combination with scolicidal therapy with albendazole.^9^ Surgical manipulation of the cyst must be performed carefully, as spillage of the cyst contents may cause anaphylactic shock.^10^

Shuker^11^ first reported a hydatid cyst involving the mandible.^11^ Our literature search revealed only seven cases involving the mandible. A summary of the cases reported till date is presented in Table 1.

**Table 1:** Summary of reported cases of hydatid cysts involving the mandible

Reference	Age/Sex	Location	Other organ involvement
^11^	35/M	Left lower border	Not mentioned
	10/M	Coronoid and ramus	Right lobe of the liver
^12^	16/M	Right side involving the coronoid and condylar processes, ramus, and zygomatic arch	None
^13^	32/M	Left angle	None
^14^	5/M	Right angle and body	Not mentioned
^15^	35/F	Left ramus and condyle	None
^2^	15/M	Left angle and ramus	None
Present case	16/F	Left ramus	Right lobe of the liver

## CONCLUSION

The oral cavity is the gateway and mirror of overall health and well-being. Many systemic diseases have initial clinical manifestations in the oral and maxillofacial areas. Although the clinical and radiographic features of echinococcosis in the oral and maxillofacial regions are non-specific, often leading to misdiagnosis, histopathology should be considered the gold standard for diagnosis. 
