# ERRATUM: Hydatid cyst involving the mandible ramus

**DOI:** 10.4322/acr.2023.441

**Published:** 2023-07-19

Nivedhitha Maraimalai^1^https://orcid.org/0000-0003-1208-3088, Manisha Ahire Sardar^1^https://orcid.org/0000-0003-2003-1544, Kavita Wadde^2^https://orcid.org/0000-0002-0127-0876, Om Kharat^3^https://orcid.org/0000-0003-1010-691X, Shaheen Kanpurwala^4^https://orcid.org/0009-0009-1407-651X, Asha Chowdhar^2^https://orcid.org/0000-0002-3914-9262

**How to cite:** Maraimalai N, Sardar MA, Wadde K, Kharat O, Kanpurwala S, Chowdhar A. ERRATUM: Hydatid cyst involving the mandible ramus. Autops Case Rep. 2023;13:e2023441. https://doi.org/10.4322/acr.2023.441

^1^Government Dental College and Hospital, Department of Oral Pathology and Microbiology, Mumbai, India

^2^Government Dental College and Hospital, Department of Oral and Maxillofacial Surgery, Mumbai, India

^3^Government Dental College and Hospital, Department of Oral Medicine and Radiology, Mumbai, India

^4^Grant Government Medical College and Sir JJ Group of Hospitals, Mumbai, India

Due to an error in the Affiliations section, the article “Hydatid cyst involving the mandible ramus” (DOI https://doi.org/10.4322/acr.2023.437), published in Autops. Case Rep. 13, 2023, was published with an error.

On page affiliations, where the text reads:

^4^Government Dental College and Hospital, Department of General Pathology, Mumbai, India

It should read:

^4^Grant Government Medical College and Sir JJ Group of Hospitals, Mumbai, India

